# Acid leaching technology for post-consumer gypsum purification

**DOI:** 10.12688/openreseurope.16583.3

**Published:** 2024-05-01

**Authors:** Miguel Castro-Diaz, Mohamed Osmani, Sergio Cavalaro, Paul Needham, Bill Parker, Tatiana Lovato

**Affiliations:** 1 Loughborough University, Loughborough, England, LE11 3TU, UK; 2 ENVA, Nottingham, England, NG4 2JT, UK; 3 British Gypsum, Loughborough, England, LE12 6JT, UK

**Keywords:** Plasterboard waste, refurbishment, demolition, acid leaching purification, post-consumer gypsum recycling

## Abstract

**Background:**

Contaminants and water-soluble salts present in mechanically recycled gypsum from refurbishment and demolition (post-consumer) plasterboard waste limit its use as a secondary raw material in plasterboard manufacturing. This research addresses this limitation, developing a novel acid leaching purification technology combined with an improved mechanical pre-treatment for post-consumer gypsum valorization.

**Methods:**

Laboratory-scale acid leaching purification was performed with a borosilicate beaker, hot plate, and overhead stirrer. Stuccos were produced after calcination of gypsum at 150 °C for 3 hours. Samples were characterized through X-ray fluorescence, X-ray diffraction, thermal gravimetric analysis, scanning electron microscopy and particle size analysis.

**Results:**

Acid leaching at 90 °C for 1 h using a 5 wt% sulfuric acid solution was revealed to be the optimum purification conditions. Stuccos produced from purified gypsum under optimum conditions had similar initial setting times to that of a commercial stucco but with higher water demand, which could be reduced by optimizing the calcination conditions. A magnesium-rich gypsum was precipitated from the wastewater.

**Conclusions:**

Purified post-consumer gypsum with > 96 wt% chemical purity and calcium sulfate dihydrate content was produced. The research recommends acid neutralization prior filtration, use of gypsum particles < 2 mm in size, and stirring speed of 50 rpm to reduce the economic and environmental impacts of the acid leaching purification process at industrial scale. The magnesium-rich gypsum could potentially be marketed as soil fertilizer.

## Plain language summary

Plasterboard is a construction material constituted by a gypsum core sandwiched between two paper liners. Plasterboard waste generated in refurbishment and demolition projects, which is known as post-consumer plasterboard waste, contains contaminants such as mortar, plastics, foil and wood, and impurities originating from additives introduced during plasterboard manufacturing and from plasterboard finishings, such as paint and wallpaper. These contaminants and impurities cannot be removed with current mechanical treatments used in plasterboard recycling plants, and post-consumer plasterboard waste is disposed of in landfills. A novel acid leaching purification process was developed here and combined with a modified mechanical treatment that produces high purity recycled gypsum from post-consumer plasterboard waste that fulfils the requirements for plasterboard manufacturing. This new plasterboard recycling technology will prevent plasterboard waste landfilling and increase the recycled content in new plasterboards. As a result, mineral gypsum use in new plasterboards will be reduced, which will lower the environmental impact associated to mineral gypsum extraction and transportation.

## Introduction

### Plasterboard waste generation

Plasterboard waste is generated during construction, refurbishment, and demolition projects. Actual amounts of plasterboard waste generated in the EU and respective Member States are unavailable, but it is estimated that 2.35 million tons of plasterboard waste are produced annually in Europe, with an extra 0.6 million tons produced during plasterboard manufacturing and installation ^1, 2^. Current plasterboard recycling plants rely on several mechanical processes, namely manual segregation, grinding, sieving, and magnetic separation, that remove paper, concrete, foam, paint, plastics, wood, ceramics, glass, and ferrous metals from gypsum. These recycling processes are almost exclusively aimed at the recovery of pre-consumer plasterboard waste, namely onsite-plasterboard offcuts ^3^. On the other hand, post-consumer plasterboard waste from refurbishment and demolition projects has high levels of contaminants that can damage equipment in current recycling plants ^4^, which hinder its operational recovery and recycling. This highlights the lack of suitable purification technologies for post-consumer plasterboard waste.

### Post-consumer gypsum recycling

Although gypsum (CaSO _4_.2H _2_O) is a material that can be recycled indefinitely through calcination-rehydration cycles and at a lower cost than landfilling ^1^, it must meet several requirements for plasterboard manufacturing. The British Standard Institute PAS 109 recommended CaSO _4_.2H _2_O contents in recycled gypsum of > 85 wt% ^5^. One of the main challenges to produce recycled gypsum from post-consumer plasterboard waste is attributed to the difficulty to achieve consistent CaSO _4_.2H _2_O contents > 92 wt% via current mechanical recycling processes ^3^. Indeed, CaSO _4_.2H _2_O content of recycled gypsum from post-consumer plasterboard waste is typically in the range 75-90 wt% ^6^.

Water-soluble phosphorus, chloride, magnesium, sodium, and potassium salts could also be present in mechanically recycled gypsum, which migrate to the paper-gypsum interface during plasterboard drying and affect paper bonding strength ^4^. The GtoG project established that the content of these salts in recycled gypsum should be < 0.02 wt% ^6^, but the uncertain quantity of salt content in recycled gypsum from post-consumer plasterboard waste restricts its utilization in plasterboard manufacturing ^4^. This was related to high water-soluble salt contents, which was ascribed to restraining residual paper in recycled gypsum ^3^.

Acid leaching is a purification process that has been used almost exclusively for the removal of toxic heavy metals and radioactive nuclides in phosphogypsum, which is obtained as a by-product during phosphoric acid production ^7– 13^. This purification process was performed with sulfuric acid, H _2_SO _4_, because is relatively cheap and yields more gypsum after neutralization with calcium oxide or calcium carbonate ^14, 15^. Higher temperature and H _2_SO _4_ content, and longer residence time were found to increase acid leaching purification efficiency ^16^.

### Aims of this work

Our preliminary work ^17^ was the first to demonstrate that an improved mechanical pre-treatment followed by acid leaching purification at 90 °C for 1 h using a 5 wt% H _2_SO _4_ solution can achieve purity levels > 96 wt% in gypsum from refurbishment plasterboard waste. The main aims of the current work were to (1) demonstrate that the improved mechanical pre-treatment together with the novel acid leaching purification process can produce purified post-consumer gypsum with consistent > 96 wt% purity, which is the current maximum purity in recycled gypsum ^3^; (2) evaluate the performance of calcined purified gypsum samples (stuccos); and (3) propose an industrial-scale acid leaching purification plant design to minimize environmental and economic impacts. Two approaches for post-consumer gypsum purification were evaluated: i) acid leaching purification followed by filtration, washing, and drying, to maximize the efficiency of the acid leaching purification process; and ii) acid leaching followed by neutralization, filtration and drying, to reduce the economic and environmental impacts of the acid leaching purification process. 

## Materials and methods

### Plasterboard waste sourcing

**Figure 1.**
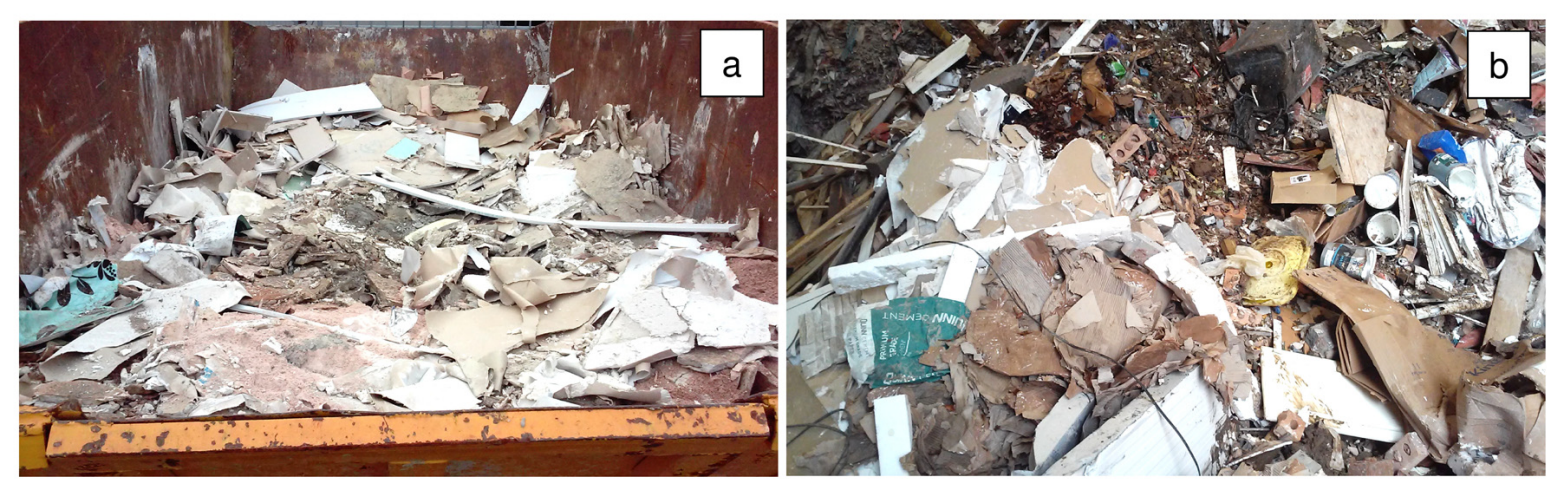
Plasterboard waste sample sourcing. ( **a**) Refurbishment plasterboard waste (RPW): household waste and recycling center, Nottingham, United Kingdom. ( **b**) Demolition plasterboard waste (DPW): recycling site, Nottingham, United Kingdom.

Refurbishment plasterboard waste (RPW) was sourced from a household waste and recycling center in Nottingham (United Kingdom), and demolition plasterboard waste (DPW) was obtained from a recycling site in Leicester (United Kingdom). The collected refurbishment and demolition plasterboard waste samples are shown in Figure 1. 

Approximately 30 kg of each waste was collected in plastic bags to be transported to the laboratory. Then, the wastes were manually segregated in the laboratory to determine their total contaminant content. Contaminants comprising paper, mortar, plastics, foam, wood, and glass were found in the collected plasterboard waste samples. RPW contained 10 wt% of contaminants whilst DPW contained 23 wt% of contaminants. 

### Reference materials and chemicals sourcing

Mineral gypsum (MG) and commercial stucco (CS) currently used in plasterboard manufacturing were provided as fine powders by a UK plasterboard supplier. MG was used for comparison purposes and to define the criterion for the calculation of the chemical purity of the samples through X-ray fluorescence. CS was produced in a continuous calciner at 150 °C and was used as reference to evaluate stuccos obtained from gypsum from post-consumer plasterboard waste before and after acid leaching purification. Sulfuric acid (H _2_SO _4_, Fisher Chemicals, certified analytical reagent, minimum purity 95 vol%) and distilled or purified water were used to prepare the H _2_SO _4_ solutions to carry out the acid leaching tests. Calcium hydroxide powder (Ca(OH) _2_, Acros Organics, ACS reagent, purity > 95 wt%) was used in neutralization and wastewater treatment tests.

### Post-consumer gypsum preparation

**Figure 2.**
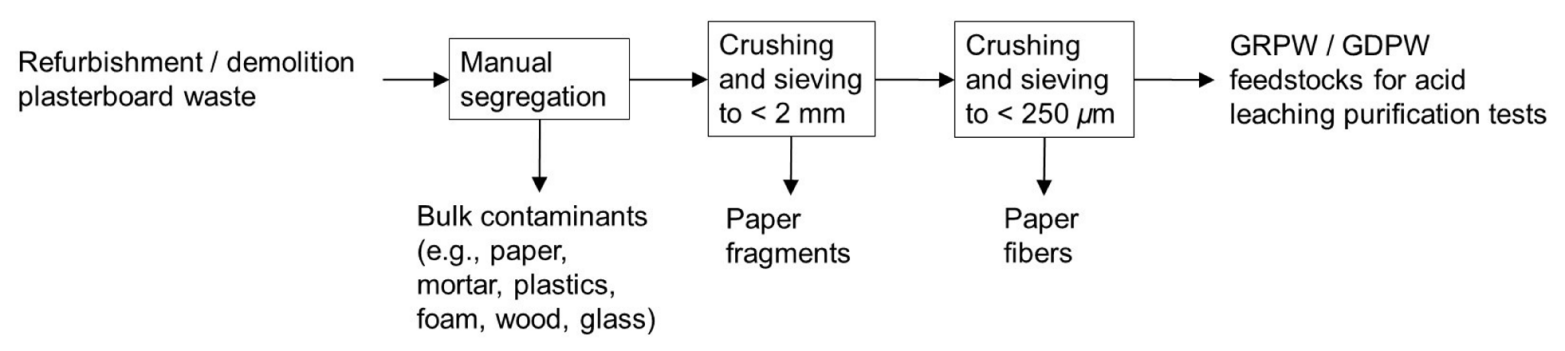
Schematic representation of the improved mechanical pre-treatment. This mechanical pre-treatment produced the GRPW or GDPW feedstocks for acid leaching purification tests.

Post-consumer gypsum with particle sizes < 250 *µ*m was prepared through crushing and sieving of refurbishment and demolition plasterboard wastes. Crushing and sieving allowed for the removal of paper fragments and fibers, and particle sizes < 250 *µ*m were chosen to increase acid leaching purification efficiency. Two sieves with apertures of 2 mm and 250 *µ*m and a receiver tray were used at the sieving stage. The diameter of the sieves and tray was 300 mm, and the sieves conformed to standards ISO 3310-1 and BS 410-1. Initially, RPW and DPW samples were broken down into fragments < 4 cm in size. These fragments were then crushed manually with porcelain mortar and pestle, and the crushed material was sieved to obtain < 2 mm particle sizes and remove paper fragments. Afterwards, the material passing was crushed again using the same procedure described above and sieved to obtain < 250 *µ*m particle sizes and remove paper fibers, which represented < 0.5 wt% of the obtained post-consumer gypsum. The post-consumer gypsum obtained from refurbishment and demolition plasterboard waste samples will be referred to as GRPW and GDPW, respectively. The procedure to obtain GRPW and GDPW is displayed in Figure 2. Two batches of GRPW (1.5 kg each) and two batches of GDPW (1.5 kg each) were used as feedstocks for acid leaching purification tests. Gypsum particles < 2 mm in size obtained from refurbishment plasterboard waste were also used in one acid leaching purification test.

### Laboratory-scale acid leaching post-consumer gypsum purification tests

In the first approach, laboratory-scale acid leaching purification tests were performed with a 500 mL Pyrex ^®^ borosilicate beaker, a hot plate, and a Camlab OS20-S LED digital overhead stirrer with a PTFE-coated crossed stirrer shaft. Tests were conducted with 100 g of either GRPW or GDPW, a gypsum/solution ratio of 1:3 wt/wt that was found optimum for acid leaching phosphogypsum purification ^8^, a gypsum slurry volume of 350 mL, and a stirring speed of either 50 or 150 revolutions per minute (rpm). Temperatures of 24 °C, 60 °C and 90 °C, H _2_SO _4_ contents of 3 wt% (0.3 M), 5 wt% (0.5 M) and 10 wt% (1.1 M), and residence times of 30 min, 1 h, 1.5 h, 2 h and 3 h were evaluated. GRPW and GDPW were added to the H _2_SO _4_ solution at room temperature and the acidic gypsum slurry was heated to the target temperature at a rate of 3–4 °C/min. The temperature of the acidic gypsum slurry was monitored with an independent thermocouple. At the end of the test, the slurry was allowed to cool down to room temperature and the purified post-consumer gypsum was recovered using a Buchner filtration kit connected to a vacuum pump. Distilled or purified water was used to wash the purified post-consumer gypsum cake. Cake washing was carried out until the color of litmus paper in contact with the filtrate indicated pH 6. Then, the purified post-consumer gypsum cake was dried in an oven at 45 °C for either 12 or 24 h depending on its water content. Drying was done at 45 °C to prevent conversion of gypsum (CaSO _4_.2H _2_O) into stucco (CaSO _4_.½H _2_O). Finally, the dried sample was crushed with ceramic mortar and pestle to produce a fine powder.

In the second approach, acid leaching purification was carried out with < 2 mm gypsum particles, stirring speed of 50 rpm and 3 wt% H _2_SO _4_ solution, and the acidic gypsum slurry was neutralized to pH 5 with Ca(OH) _2_ prior filtration. The aim of this second approach was to develop an industrial-scale acid leaching purification plant design. The same laboratory setup was used in acid leaching and neutralization steps. The pH was measured with a bench top Hanna Instruments pH meter (model HI-2211). The neutralized slurry was filtered to recover the purified post-consumer gypsum and the wastewater. The wastewater and a magnetic stir bar were placed in the same 500 mL borosilicate beaker used for acid leaching purification tests. The beaker was placed on the top plate of a magnetic stirrer and Ca(OH) _2_ powder was gradually added until a pH of 10.5 was reached. The resulting precipitate was filtered and dried at room temperature.

### Experimental techniques

The chemical composition of the gypsum samples was determined through X-ray fluorescence (XRF). XRF analyses were conducted with an Orbis micro-XRF spectrometer. Pellets for XRF characterization were prepared by blending 0.8 g of gypsum powder with 0.2 g of boric acid powder (binder). Then, this blend was placed in a die and piston of 5 mm in diameter and compacted in a manual hydraulic press applying 10 tons of force. XRF data were acquired under vacuum in five regions of the pellet using a voltage of 30 kV, current of 0.4 mA, amplifier time of 1.6 µs and acquisition time of 120 s. The weight percentages of SO _3_, CaO, SiO _2_, Al _2_O _3_, Fe _2_O _3_, MnO, MgO, P _2_O _5_, K _2_O, Na _2_O, Ni _2_O _3_, SrO and Cl were recorded. Previous research identified MgO, P _2_O _5_, K _2_O, Na _2_O and Cl compounds present in gypsum as detrimental for plasterboard manufacturing ^4, 7^. MG was also used as reference to establish the methodology for the calculation of gypsum’s chemical purity through XRF. MnO content in GRPW and GDPW was higher than in MG (Table S1 in *Extended data* ^18^), whereas Ni _2_O _3_ and SrO contents were mostly below the detection limit of the XRF spectrometer (< 0.1 wt%). In this work, MnO, Ni _2_O _3_ and SrO were assumed to be impurities and the chemical purity of gypsum was considered as the sum of SO _3_, CaO, SiO _2_, Al _2_O _3_ and Fe _2_O _3_ contents ( Equation 1). The CaSO _4_ content (i.e., sum of CaO and SO _3_ contents) was also determined to differentiate between gypsum samples with similar chemical purity values.

*Chemical purity (wt%)* = *SO*_3_ + *CaO* + *SiO*_2_ + *Al*_2_ *O*_3_ + *Fe*_2_ *O*_3_           (1)

The contents of gypsum (CaSO _4_.2H _2_O), bassanite or stucco (CaSO _4_.½H _2_O), quartz (SiO _2_) and calcite (CaCO _3_) in the gypsum samples and the contents of CaSO _4_.2H _2_O, CaSO _4_.½H _2_O and anhydrous CaSO _4_ in the stuccos were determined through X-ray diffraction (XRD). XRD patterns were obtained using a Bruker D2 Phaser X-ray diffractometer, fitted with a 1-dimensional Lynxeye detector, and using Ni filtered Cu Kα radiation run at 30 kV and 10 mA. Patterns were recorded from 10–100° 2θ, using a step size of 0.02, and were analyzed with DIFFRAC.EVA diffraction software. ICDD-PDF numbers 74-1433, 33-0310, 83-0437, 05-0490 and 05-0586 were used for the semi-quantitative and qualitative analysis of CaSO _4_.2H _2_O, CaSO _4_.½H _2_O, CaSO _4_, SiO _2_ and CaCO _3_, respectively. XRD analysis of the precipitates obtained from the wastewater was also carried out. Gypsum (CaSO _4_.2H _2_O), brucite [Mg(OH) _2_], kieserite (MgSO _4_.H _2_O) and portlandite [Ca(OH) _2_] were identified and quantified using ICDD-PDF numbers 74-1433, 07-0239, 70-2156 and 04-0733, respectively.

GRPW and GDPW before and after acid leaching purification tests were characterized through thermal gravimetric analysis (TGA) and derivative thermogravimetry (DTG). MG was also characterized for comparison purposes. TGA/DTG profiles were recorded with a TA Q5000IR thermogravimetric analyzer (TA Instruments Inc., US). An amount of 20 mg was placed in a sealed aluminum pan with a pierced lid and heated from 40 °C to 250 °C using a heating rate of 5 °C/min. A nitrogen flow rate of 20 mL/min was applied to the balance throughout the test. Stoichiometrically, pure CaSO _4_.2H _2_O is constituted by 20.93 wt% of H _2_O. Therefore, the theoretical CaSO _4_.2H _2_O content in the gypsum samples was calculated by multiplying the weight loss at 240 °C by a factor of 4.778.

The particle size distribution of GRPW and GDPW feedstocks used in acid leaching purification tests was determined with a Malvern Mastersizer 3000 with Hydro EV dispersion unit and wet measurement cell, using isopropanol as dispersant. Five determinations were performed with each sample and average values were calculated. A Zeiss EVO 50 scanning electron microscopy (SEM) instrument was used to investigate the crystal morphology of GRPW before and after acid leaching. First, the samples were sprinkled onto a carbon tab attached to the SEM stub, and then, coated in gold to reduce charging. SEM images were acquired using a magnification of ×500.

GRPW and GDPW feedstocks and purified samples with particles < 150 *µ*m in size were calcined at 150 °C for 1 h in a stationary oven to produce stuccos. Three 1-hour calcination steps were required to reduce the CaSO _4_.2H _2_O content in the samples to < 2 wt%. An ELE Automatic Vicat apparatus that complies with British standard EN 13279-2:2014 was used to determine the initial setting time of each stucco. A water/stucco ratio of 0.7 wt/wt that was adopted in similar studies ^19– 22^ was used. Distilled water and stucco were mixed manually for 90 s prior testing. Preliminary Vicat tests performed with 200 g, 300 g and 500 g of CS showed that good data reproducibility was only achieved when using 200 g. Then, each mixture containing 200 g of stucco was placed inside a Vicat conical ring of 40 mm in height, upper internal diameter of 60 mm and lower internal diameter of 70 mm. The Vicat conical ring was mounted on a round glass plate of 150 mm diameter and 6 mm thickness. The initial setting times were recorded when the needle was 22 mm above the glass plate (or 18 mm penetration), as specified by British standard EN 13279-2:2014. All Vicat tests were performed in triplicate and average values were calculated.

## Results and discussion

### Improved mechanical pre-treatment

The manual segregation step of the improved mechanical pre-treatment ( Figure 2) reduced the contaminant content in batch 1 of RPW from 10 wt% to 7 wt% and in batch 1 of DPW from 23 wt% to 15 wt%. Subsequent crushing and sieving of plasterboard fragments to produce gypsum particles < 250 *µ*m in size reduced the contaminant content in post-consumer gypsum to 4–6 wt%.

Table S1 in *Extended data* ^18^ shows the chemical composition and chemical purity of the two different batches of GRPW and GDPW studied in this work and the composition of MG. The chemical purity values of batch 1 and batch 2 of GRPW calculated using Equation 1 were respectively 95.9 wt% and 94.9 wt%. Likewise, the chemical purity values of batch 1 and batch 2 of GDPW were respectively 96.0 wt% and 94.7 wt%. These results show that the difference in the chemical purity of batch 1 and batch 2 of GRPW and GDPW was 1–1.3 wt%. Moreover, the chemical purity values of these batches were comparable to that of MG (95.1 wt%). The CaSO _4_ contents of GRPW and GDPW (94.3 wt% and 93.2 wt%) were higher than in MG (89.9 wt%). The main impurity in GRPW and GDPW was phosphorus (P _2_O _5_ ≥ 2 wt%). 

**Figure 3.**
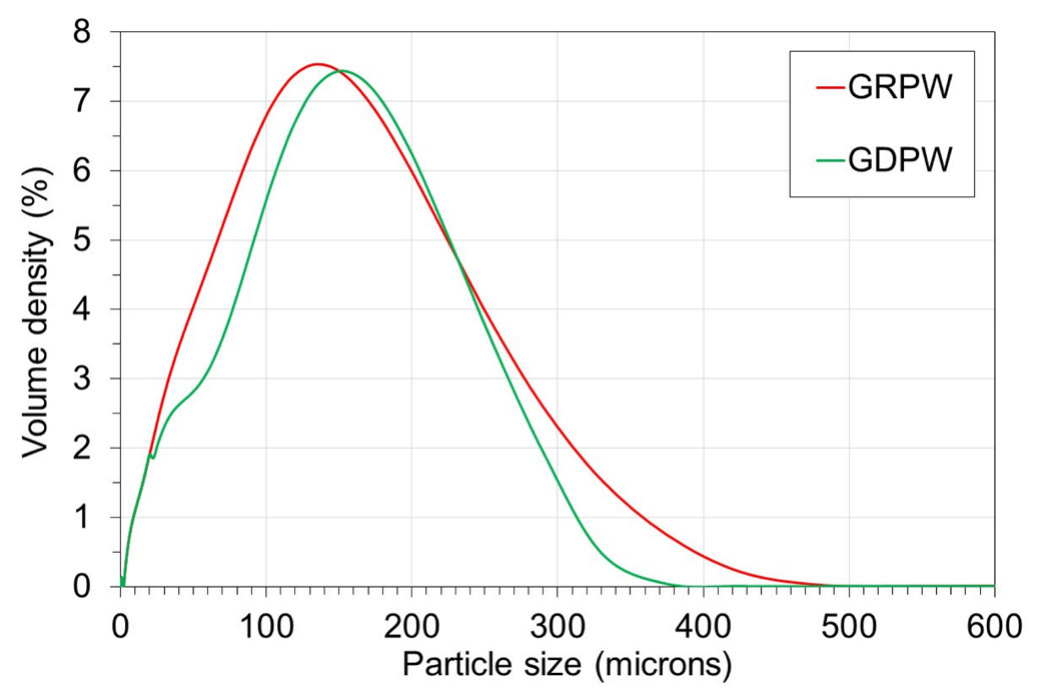
Particle size distribution of batch 1 of gypsum from refurbishment from plasterboard waste (GRPW) and batch 1 of gypsum from demolition plasterboard waste (GDPW) used as feedstocks in acid leaching purification tests.

The particle size distribution profiles of GRPW and GDPW are presented in Figure 3. The particle size distribution profiles of GRPW and GDPW were similar, with the maximum particle volume densities at around 135 *µ*m and 150 *µ*m, respectively. The presence of particles > 250 *µ*m in size in both samples could be due to agglomeration of gypsum particles and/or presence of non-spherical particles (e.g., paper fibers).

### Acid leaching post-consumer gypsum purification

**Figure 4.**
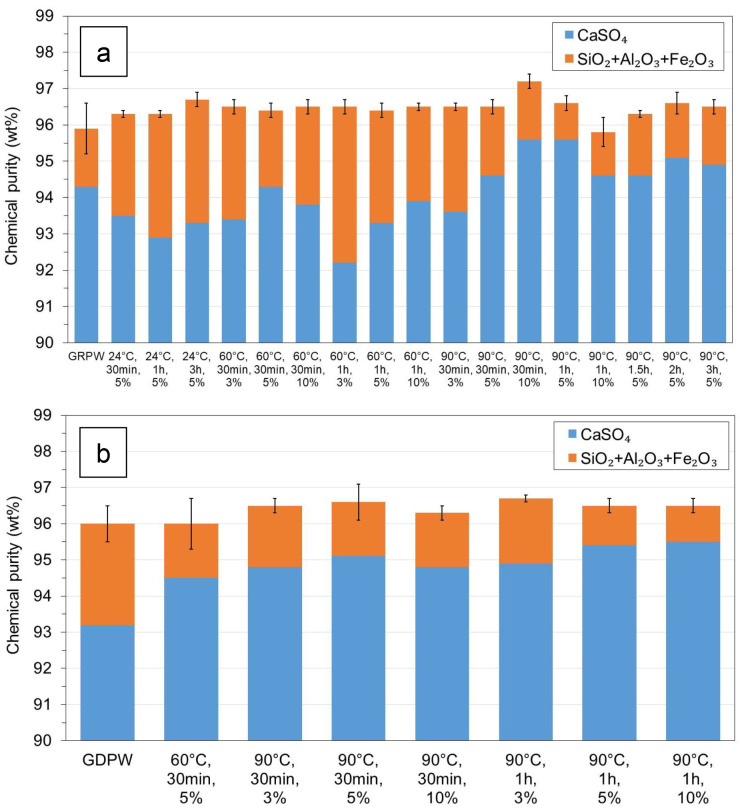
Chemical purity of post-consumer gypsum before and after acid leaching using different temperatures, residence times and H _2_SO _4_ contents. ( **a**) Batch 1 of gypsum from refurbishment plasterboard waste (GRPW). ( **b**) Batch 1 of gypsum from demolition plasterboard waste (GDPW).

The results from laboratory-scale acid leaching purification tests with batch 1 of GRPW and batch 1 of GDPW using different temperatures, residence times and H _2_SO _4_ contents are presented in Figures 4a and 4b, respectively. The actual data can be found in Table S2 in *Extended data* ^18^.

In the case of GRPW ( Figure 4a), there were no significant differences in the chemical purity of the purified samples (around 96.5 wt%) and, in general, these values were 0.5–1.0 wt% higher than that of the GRPW feedstock. These results might suggest that acid leaching purification at 60 °C for 30 min using a 3 wt% H _2_SO _4_ solution would be sufficient to produce purified GRPW with chemical purity > 96 wt%. However, the CaSO _4_ contents in purified gypsum were usually lower when acid leaching was performed at 60 °C than at 90 °C, which could be explained by the Ostwald ripening process. This process consists of the initial dissolution of small CaSO _4_.2H _2_O crystals followed by deposition and recrystallization of dissolved CaSO _4_ on the surface of larger crystals. The Ostwald ripening process was observed by Zheng *et al*. ^11^ during acid leaching of a gypsum waste under hydrothermal conditions at 100–120 °C. The CaSO _4_ content in batch 1 of GRPW increased from 94.3 wt% in the feedstock to 95.6 wt% when acid leaching purification was carried out at 90 °C, either for 30 min using a 10 wt% H _2_SO _4_ solution or for 1 h using a 5 wt% H _2_SO _4_ solution. Therefore, it is thought that acid leaching under these conditions favored the deposition and recrystallization of dissolved CaSO _4_, leading to the observed increase in the CaSO _4_ content in purified GRPW. Under these two acid leaching conditions, no changes in P _2_O _5_, MnO, K _2_O and MgO contents were observed, but Na _2_O content decreased by 76% and Cl content decreased by 37%. Arguably, it would be preferable to perform acid leaching purification using a 5 wt% rather than a 10 wt% H _2_SO _4_ solution to minimize H _2_SO _4_ consumption. 

**Figure 5.**
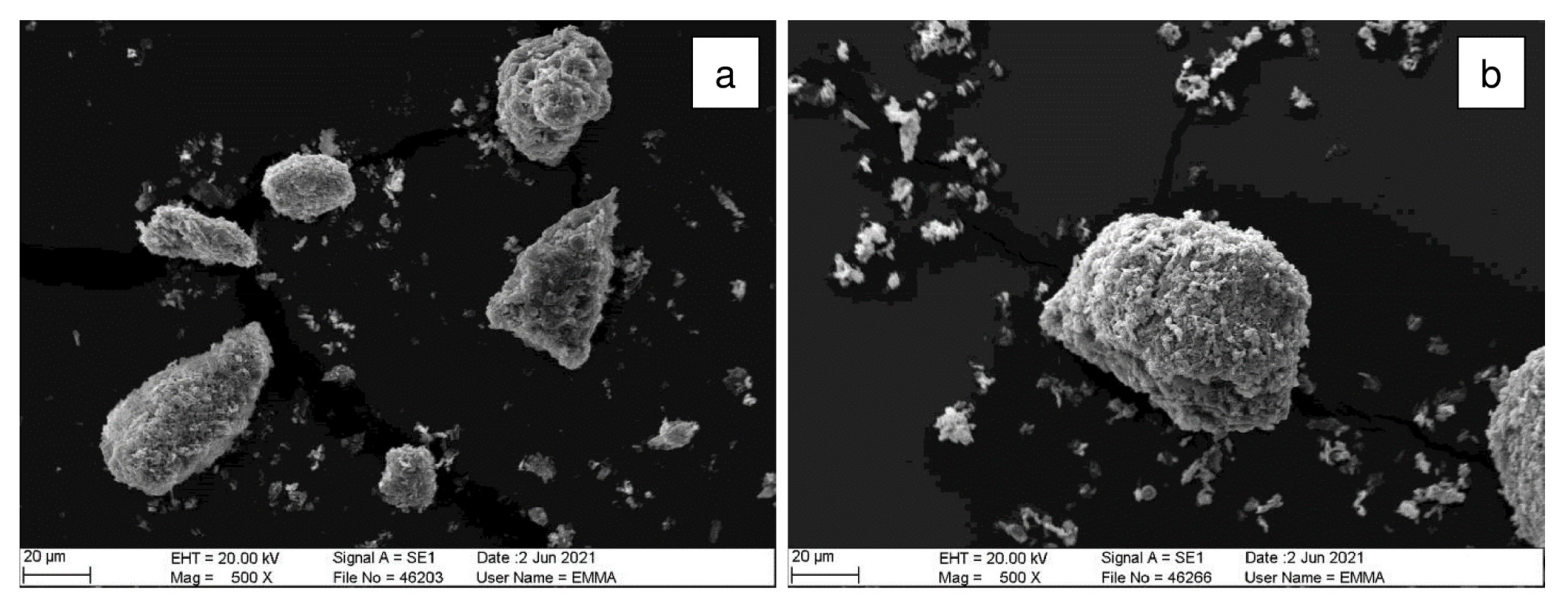
SEM images. ( **a**) GRPW feedstock. ( **b**) GRPW after acid leaching at 90 °C for 1 h using a 5 wt% H _2_SO _4_ solution.

SEM images of the crystals from batch 1 of GRPW before and after acid leaching purification at 90 °C for 1 h using a 5 wt% H _2_SO _4_ solution are shown in Figure 5. GRPW feedstock appeared to be made up of agglomerations of small gypsum crystals, and acid leaching seemed to increase the gypsum crystal size. These findings concur with the hypothesis that gypsum particles underwent the Ostwald ripening process during acid leaching. 

Taking into consideration GRPW results, most acid leaching purification tests with batch 1 of GDPW were carried out at 90 °C for either 30 min or 1 h using different H _2_SO _4_ solutions. Figure 4b shows that the chemical purity did not change significantly in treated GDPW samples and were comparable to the chemical purity in treated GRPW samples (96.5 wt%). The highest CaSO _4_ content of 95.5 wt% was obtained after acid leaching purification at 90 °C for 1 h using either 5 wt% or 10 wt% H _2_SO _4_ solutions. Therefore, optimum acid leaching purification conditions were 90 °C for 1 h using a 5 wt% H _2_SO _4_ solution, which resulted in an increase of 0.5–0.7 wt% in the chemical purity and an increase of 1.3–2.2 wt% in the CaSO _4_ content of GRPW and GDPW.

The CaSO _4_.2H _2_O content in GRPW and GDPW before and after acid leaching purification under optimum conditions of 90 °C for 1 h using a 5 wt% H _2_SO _4_ solution was determined semi-quantitatively through XRD and calculated theoretically through TGA. XRD peaks positioned at 11.6°, 20.7°, 23.4°, 29.1°, 31.1°, 33.3° and 43.3° are associated to CaSO _4_.2H _2_O, whereas peaks positioned at 14.6°, 25.6°, 29.7°, 31.7°, 32.9° and 49.1° are associated to CaSO _4_.½H _2_O ^23, 24^. The XRD spectra of all samples were dominated by the presence of CaSO _4_.2H _2_O (Figure S1 in *Extended data* ^18^). Peaks associated to CaSO _4_.½H _2_O were absent in GRPW, GDPW and MG spectra. However, there were small peaks associated to CaCO _3_ and SiO _2_. 

**Figure 6.**
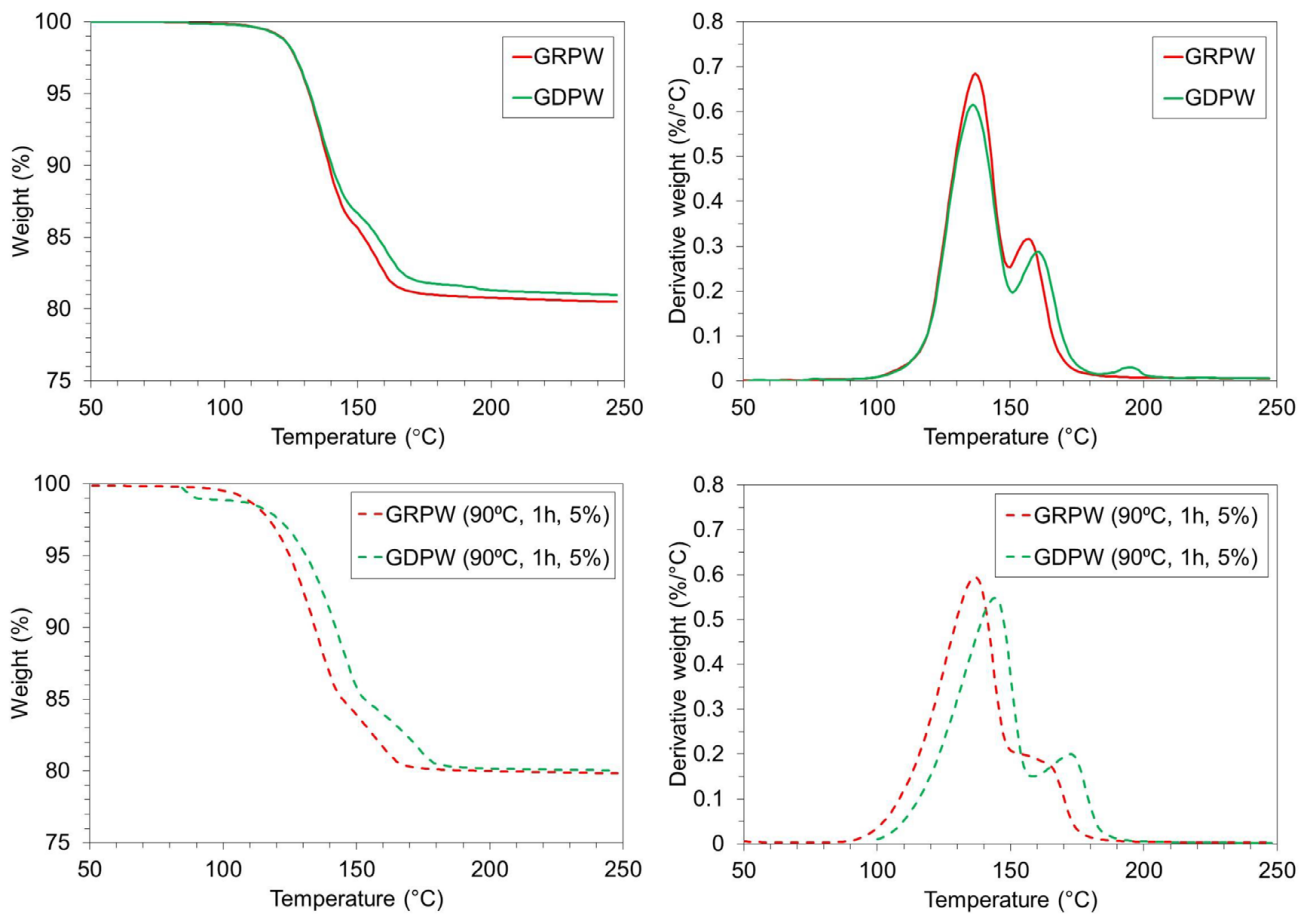
TGA and DTG profiles. Batch 1 of gypsum from refurbishment plasterboard waste (GRPW) and batch 1 of gypsum from demolition plasterboard waste (GDPW) before (top) and after (bottom) acid leaching at 90 °C for 1 h using a 5 wt% H _2_SO _4_ solution.

Figure 6 shows TGA and DTG results for GRPW and GDPW before and after acid leaching purification under optimum conditions. Acid leaching purification increased the weight loss in GRPW and GDPW by around 0.5 wt%, which could be attributed to the removal of chemical impurities and increase in CaSO _4_ content compared to the feedstocks (Figures 4a and 4b). The first devolatilization peak at around 135 °C in the DTG profiles (peak 1) is associated to water removal from CaSO _4_.2H _2_O as it converts into CaSO _4_.½H _2_O. The second devolatilization peak (peak 2) corresponds to water removal from CaSO _4_.½H _2_O to produce anhydrous CaSO _4_. The DTG profiles of GRPW and GDPW show only small differences.

The CaSO _4_.2H _2_O content in GRPW and GDPW before and after acid leaching purification under optimum conditions was determined with XRD and TGA data ( Table 1). The CaSO _4_.2H _2_O content in GRPW and GDPW determined through XRD was usually higher than that calculated from TGA data. This could be rationalized by the impact of paper fibers in TGA results. 

**Table 1.** Mineral composition and CaSO _4_.2H _2_O content calculated respectively through XRD and TGA for batch 1 of GRPW and batch 1 of GDPW before and after acid leaching purification at 90 °C for 1 h using a 5 wt% H _2_SO _4_ solution.

Sample	XRD				TGA
	CaSO _4_.2H _2_O (wt%)	CaSO _4_.½H _2_O (wt%)	SiO _2_ (wt%)	CaCO _3_ (wt%)	CaSO _4_.2H _2_O (wt%)
GRPW (feedstock)	95	Trace	1	4	93
GRPW (purified)	98	Trace	1	1	96
GDPW (feedstock)	91	4	1	4	91
GDPW (purified)	97	Trace	2	1	95

XRD data show that acid leaching purification increased the CaSO _4_.2H _2_O content of GRPW and GDPW above 96 wt%, and decreased the CaCO _3_ content, as it reacted with H _2_SO _4_ to produce CaSO _4_.2H _2_O and CO _2_ ( Equation 2). Therefore, it is thought that the increase in CaSO _4_.2H _2_O content was not only caused by the removal of impurities but also by the reaction of CaCO _3_ with H _2_SO _4_.

*CaCO*_3( *s*)_ + *H*_2_ *SO*_4( *l*)_ + *H*_2_ *O*_( *l*)_ → *CaSO*_4_. *2H*_2_ *O*_( *s*)_ + *CO*_2( *g*)_           (2)

TGA data show a 3–4 wt% increase in CaSO _4_.2H _2_O content in GRPW and GDPW after acid leaching purification. Acid leaching purification of GDPW did not produce purified post-consumer gypsum with > 96 wt% CaSO _4_.2H _2_O content mainly because the CaSO _4_.2H _2_O content in the GDPW feedstock was low (91 wt%). TGA results suggest that the improved mechanical pre-treatment of post-consumer plasterboard waste must produce GRPW and GDPW with > 92 wt% CaSO _4_.2H _2_O content for the acid leaching purification process to be effective and produce purified post-consumer gypsum with > 96 wt% CaSO _4_.2H _2_O content.

Overall, the CaSO _4_.2H _2_O content of GRPW and GDPW feedstocks ranged between 91–95 wt% and the CaSO _4_.2H _2_O content of purified GRPW and purified GDPW was ≥ 96 wt% when the CaSO _4_.2H _2_O content of the feedstocks was > 92 wt%. In addition, most CaCO _3_ present in GRPW and GDPW feedstocks reacted with H _2_SO _4_ to produce CaSO _4_.2H _2_O ( Equation 2).

### Properties of stuccos from post-consumer gypsum

The composition, water demand and initial setting times of the stuccos from batch 2 of GRPW (S-GRPW) and batch 2 of GDPW (S-GDPW) before and after optimum acid leaching purification conditions were determined ( Table 2). The results for the commercial stucco (CS) and the stucco obtained from MG (S-MG) are also presented for comparison purposes. The stuccos from purified GRPW and GDPW had CaSO _4_.½H _2_O contents between 68.7 wt% and 71.8 wt%, which were comparable to that of CS (68.5 wt%) but much higher than that of S-MG (64.1 wt%). The initial setting times of the stuccos from purified GRPW and GDPW were also similar to that of CS (4 min and 40 s), whereas the initial setting time of S-MG was shorter (4 min). The long setting time of the stucco obtained from the GRPW feedstock (12 minutes) could be due to its low CaSO _4_.½H _2_O content (59 wt%) and/or high anhydrous CaSO _4_ content (39.1 wt%) compared to the other stuccos. However, further research would be required to get a better understanding of this finding, which is outside the scope of this work. A water/stucco ratio of 0.7 wt/wt was used with CS and S-MG. However, a water/gypsum ratio of 1.2 wt/wt was required to achieve normal consistency with S-GRPW and S-GDPW before and after acid leaching purification. Pedreño-Rojas *et al*. ^25^ found that calcination at 150 °C for 3 h of a gypsum waste powder converted CaSO _4_.2H _2_O into CaSO _4_.½H _2_O but required a water/plaster ratio of 1.0 wt/wt, which was higher than the 0.55 wt/wt ratio of a commercial plaster. Bumanis *et al*. ^26^ also found that mechanically recycled gypsum from construction and demolition plasterboard waste had higher water demand than a commercial gypsum. The water demand of the stuccos from purified GRPW and GDPW could be reduced with a retardant such as citric acid ^27^. Alternatively, the calcination conditions could be optimized to reduce the water demand of these stuccos ^28^. Optimization of the calcination conditions would be preferable since citric acid is expensive and was found to impact the compressive strength of hardened gypsum plaster ^27, 29^.

**Table 2.** Composition, water demand and initial setting times of the commercial stucco (CS), stucco from the mineral gypsum (S-MG) and stuccos from batch 2 of gypsum from refurbishment plasterboard waste and batch 2 of gypsum from demolition plasterboard waste (S-GRPW and S-GDPW) before and after acid leaching at 90 °C for 1 h using a 5 wt% H _2_SO _4_ solution.

Sample	CaSO _4_.2H _2_O (wt%)	CaSO _4_.½H _2_O (wt%)	CaSO _4_ (wt%)	Water/stucco ratio (wt/wt)	Initial setting time (mm:ss)
CS (reference 1)	2.0	68.5	29.5	0.7	04:40
S-MG (reference 2)	2.3	64.1	33.6	0.7	04:00
S-GRPW (feedstock)	1.9	59.0	39.1	1.2	12:00
S-GRPW (purified)	1.2	71.8	27.0	1.2	04:25
S-GDPW (feedstock)	2.0	63.2	34.8	1.2	04:45
S-GDPW (purified)	1.9	68.7	29.4	1.2	04:40

### Industrial-scale acid leaching post-consumer gypsum purification plant design

An industrial-scale acid leaching purification plant design for post-consumer gypsum is proposed in this work based on the evaluation of some considerations to reduce economic and environmental impacts. These considerations were:

1. neutralization of the acidic gypsum slurry with Ca(OH) _2_ to pH 5 prior filtration rather than washing to avoid the use of expensive corrosion-resistant pumps and filtration equipment, reduce water consumption and prevent the precipitation of impurities;
2. stirring speed of 50 rpm rather than 150 rpm in the acid leaching step to avoid the use of expensive high-torque agitators;
3. use of gypsum particles < 2 mm rather than < 250 *μ*m in size because the former can be easily produced with current milling equipment without the need of drying; and
4. use of 3 wt% H _2_SO _4_ solution for 2 h to reduce H _2_SO _4_ consumption in the acid leaching step and Ca(OH) _2_ consumption in the neutralization step.

**Figure 7.**
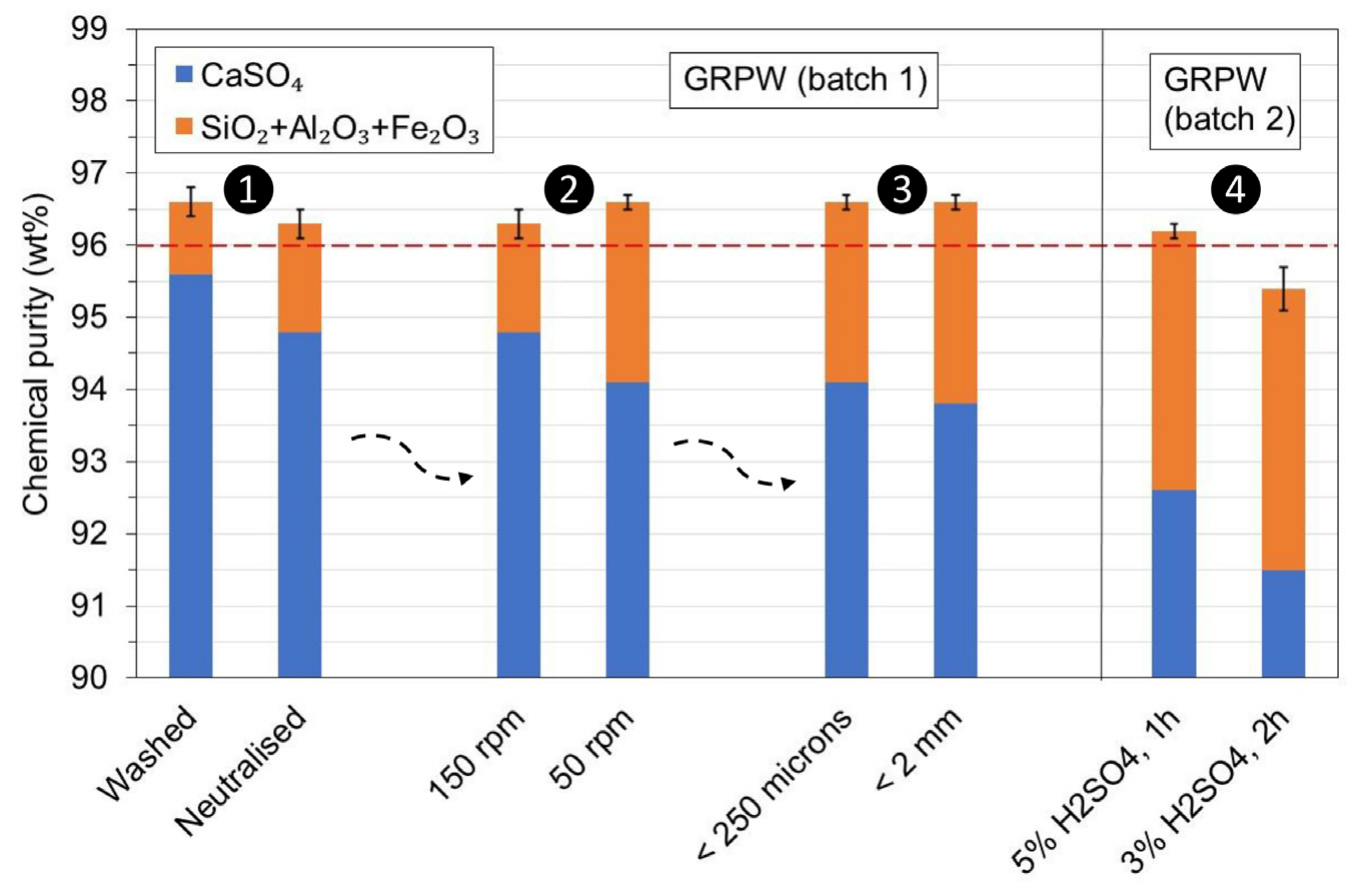
Chemical purity of purified batches 1 and 2 of gypsum from refurbishment plasterboard waste (GRPW) obtained with the four considerations. The red line represents the target chemical purity value for purified post-consumer gypsum.

These considerations were evaluated in the laboratory with batches 1 and 2 of GRPW ( Figure 7). Batch 1 of GRPW was used to evaluate the first, second and third considerations in sequential order, and the results from the first test (washed) were obtained under optimum conditions. Batch 2 of GRPW was used to evaluate the fourth consideration using particle sizes < 250 *μ*m at 90 °C with stirring at 50 rpm, followed by neutralization.

**Figure 8.**
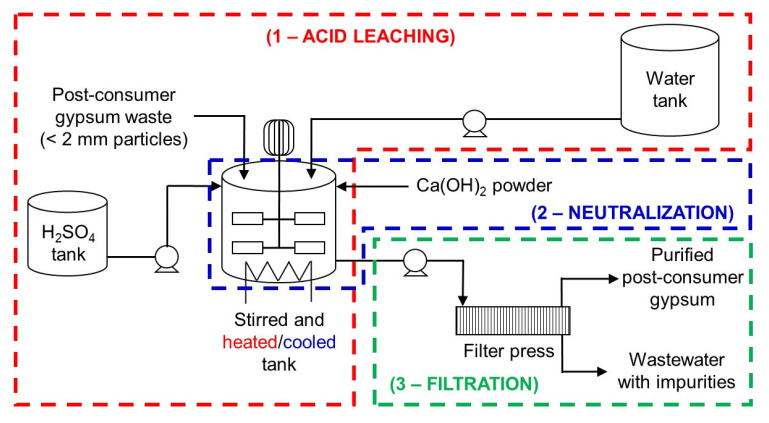
Proposed industrial-scale post-consumer gypsum purification plant design. The plant consists of three processes: acid leaching, neutralization and filtration.

Neutralization of the acidic gypsum slurry to pH 5 prior filtration decreased the chemical purity of purified GRPW by 0.3 wt% and its CaSO _4_ content by 0.8 wt% compared to the purified GRPW obtained after washing. A reduction in the stirring speed from 150 rpm to 50 rpm increased the chemical purity of purified GRPW by 0.3 wt% but reduced its CaSO _4_ content by 0.7 wt%. The chemical purity was approximately 96.5 wt% regardless of whether gypsum particle sizes < 2 mm or < 250 *μ*m were used, but the CaSO _4_ content decreased by 0.3 wt% with gypsum particle sizes < 2 mm. The reduction in H _2_SO _4_ content from 5 wt% to 3 wt% and increase in residence time from 1 h to 2 h decreased the chemical purity by 0.8 wt% and the CaSO _4_ content by 1.1 wt%. These findings suggest that only the fourth consideration would have a significant negative impact on the chemical purity and CaSO _4_ content of purified post-consumer gypsum. Hence, the proposed industrial-scale post-consumer gypsum purification plant design ( Figure 8) consists of three processes or steps: 1) acid leaching; 2) neutralization; and 3) filtration. It is envisaged that the integration of this acid leaching purification plant in current plasterboard waste recycling sites will increase capital costs (e.g., gypsum milling equipment, storage tanks, reaction tanks, filter press, pumps) and operating costs (e.g., energy for agitator, pumps and heating system, sulfuric acid, calcium hydroxide). On the other hand, post-consumer plasterboard waste recycling through the acid leaching purification process would eliminate landfilling, increase circularity of new plasterboards, and reduce mineral gypsum extraction and consumption. From an economic standpoint, the recovery and valorization of soluble impurities in the process wastewater could compensate for the operating costs of the acid leaching purification plant. In laboratory-scale trial tests, the pH of the wastewater obtained after neutralization was raised from 5 to 10.5 with the addition of Ca(OH) _2_. Magnesium-rich gypsum constituted by 79.0–87.7 wt% CaSO _4_.2H _2_O, 5.2–8.9 wt% magnesium hydroxide, Mg(OH) _2_, 6.1–9.6 wt% magnesium sulfate dihydrate, MgSO _4_.2H _2_O, and 1.0-4.0 wt% Ca(OH) _2_ was precipitated. This magnesium-rich gypsum would be classified as an inorganic secondary nutrient fertilizer and could be commercialized as a source of Ca and Mg for oil palm growth and as soil ameliorant ^30– 32^. From an environmental standpoint, CO _2_ would be produced by reaction of H _2_SO _4_ with the CaCO _3_ from contaminants such as Portland cement ^33^. The maximum CaCO _3_ content in GRPW and GDPW was 4 wt% (Table S1 in *Extended data* ^18^). Using Equation 2, the theoretical amount of CO _2_ that would be produced is 17.6 kg per ton of gypsum processed. Considering that gypsum represents 95 wt% of standard plasterboards ^34– 36^, 16.7 kg of CO _2_ would be generated per ton of plasterboard processed. On the other hand, increasing the post-consumer plasterboard waste recycling rate from 0 to 93.6% would cause a reduction of 0.22 kg CO _2_ equivalent per m ^2^ of plasterboard ^37^. These authors noted that the density of standard plasterboards varies from 8.4 kg/m ^2^ to 10.0 kg/m ^2^. Hence, amounts of 22.0–26.2 kg CO _2_ would be avoided per ton of plasterboard processed, which are higher than the 16.7kg of CO _2_ generated from CaCO _3_. However, life cycle costing and life cycle assessment would be required to estimate the total cost and CO _2_ emissions from the acid leching purification plant, which are outside the scope of this work.

## Conclusions

The main aims of this work were to develop an acid leaching purification process to achieve consistent chemical purity and CaSO _4_.2H _2_O contents of > 96 wt% in post-consumer gypsum; evaluate the performance of stuccos from purified post-consumer gypsum; and propose an industrial-scale acid leaching purification plant design to minimize environmental and economic impacts. Two approaches for post-consumer gypsum purification were evaluated, the first one aiming to maximize the purity level of the purified post-consumer gypsum, and the second one aiming to minimize economic and environmental impacts of the purification process. The main findings are summarized below.

1. The two-step crushing and sieving methodology of the improved mechanical pre-treatment for post-consumer plasterboard waste, first to obtain gypsum particles < 2 mm in size and then to obtain gypsum particles < 250 *µ*m in size, was very effective to remove paper fragments and fibers and produce gypsum with chemical purity > 95.5 wt% and CaSO _4_.2H _2_O content that ranged between 91–95 wt%.
2. The posterior acid leaching purification of post-consumer gypsum (< 250 *µ*m particles) at optimum conditions of 90 °C for 1 h using a 5 wt% H _2_SO _4_ solution produced purified post-consumer gypsum with chemical purity and CaSO _4_.2H _2_O content > 96.5 wt%.
3. The initial setting times of the stuccos obtained after calcination of purified samples at 150 °C for 3 h were similar to that of a commercial stucco but had higher water demand, which could be reduced by optimizing the calcination conditions.
4. The proposed industrial-scale acid leaching purification plant for post-consumer gypsum considered gypsum particles < 2 mm in size, stirring speed of 50 rpm and an acid neutralization step prior filtration to reduce the economic and environmental impacts of the process.

The combination and implementation of the improved mechanical pre-treatment and the novel acid leaching purification process developed in this work offers for the first time an effective purification technology for post-consumer gypsum waste, that will allow for higher recycled gypsum content in new plasterboards and the avoidance of post-consumer plasterboard waste landfilling. Future work will develop an optimum wastewater treatment process to maximize the recovery of impurities and produce water that can be reused in the acid leaching purification process. 

## Data Availability

Zenodo Digital Repository: Dataset for publication “Acid leaching technology for post-consumer gypsum purification”. https://doi.org/10.5281/zenodo.8279464 ^18^. This project contains the following underlying data: Dataset description.txt (abbreviations and codes of samples presented in the publication). Particle size analysis.zip (particle size distribution data files for the two acid leaching feedstocks in .txt format). SEM.zip (scanning electron microscopy images of gypsum from refurbishment plasterboard waste before and after acid leaching in .jpg format). TGA.zip (thermal gravimetric analysis raw data for gypsum from refurbishment and demolition plasterboard wastes before and after acid leaching in .xlsx format). XRD.zip (X-ray diffraction data files for samples presented in the publication in .raw, .brml and .uxd formats). XRF.zip (X-ray fluorescence data files for samples presented in the publication in .xlsx format). Zenodo Digital Repository: Dataset for publication “Acid leaching technology for post-consumer gypsum purification”. https://doi.org/10.5281/zenodo.8279464 ^18^. This project contains the following extended data: Extended data.pdf (tables and figure with direct link to the main text). Data are available under the terms of the Creative Commons Attribution 4.0 International license (CC-BY 4.0).
