# Supplementary material for: Acid leaching technology for post-consumer gypsum purification
Source: Open Res Eur. 2024 May 1;3:148. Originally published 2023 Sep 15. [Version 3] doi: 10.12688/openreseurope.16583.3 (PMC10873547; doi:10.12688/openreseurope.16583.3)
Supplement: Peer review history

# Reviewer report: Reviewer response for version 3

**DOI:** 10.21956/openreseurope.18753.r40045

**Published:** 2024-05-17

**Authors:** Tee How Tan

**Affiliations:** 1 Tunku Abdul Rahman University of Management and Technology,, Kuala Lumpur, Malaysia

Upon review, I'm pleased to note that the authors have addressed my concerns, and I recommend updating the status to "Approved."

 However, please note the following feedback:

1. Figure 3, it would be preferable to use "¬µm" instead of "microns."
2. Please check the heading for tables 1 and 2. The numbers in the compound should be subscript instead of superscript.

Is the study design appropriate and does the work have academic merit?

Yes

Is the work clearly and accurately presented and does it cite the current literature?

Yes

If applicable, is the statistical analysis and its interpretation appropriate?

Not applicable

Are all the source data underlying the results available to ensure full reproducibility?

Yes

Are the conclusions drawn adequately supported by the results?

Yes

Are sufficient details of methods and analysis provided to allow replication by others?

Yes

Reviewer Expertise:

NA

I confirm that I have read this submission and believe that I have an appropriate level of expertise to confirm that it is of an acceptable scientific standard.

# Reviewer report: Reviewer response for version 2

**DOI:** 10.21956/openreseurope.18327.r36651

**Published:** 2024-05-17

**Authors:** Tee How Tan

**Affiliations:** 1 Tunku Abdul Rahman University of Management and Technology,, Kuala Lumpur, Malaysia

The article presents a novel and promising technology for the purification of post-consumer gypsum from plasterboard waste, which could increase its recycling rate and reduce its environmental impact.

Is the study design appropriate and does the work have academic merit?

Yes

Is the work clearly and accurately presented and does it cite the current literature?

Yes

If applicable, is the statistical analysis and its interpretation appropriate?

Not applicable

Are all the source data underlying the results available to ensure full reproducibility?

Yes

Are the conclusions drawn adequately supported by the results?

Yes

Are sufficient details of methods and analysis provided to allow replication by others?

Yes

Reviewer Expertise:

Sustainable Cement & Concrete Materials, Waste Recycling in Cement & Concrete Materials, Geopolymer

I confirm that I have read this submission and believe that I have an appropriate level of expertise to confirm that it is of an acceptable scientific standard.

# Reviewer report: Reviewer response for version 2

**DOI:** 10.21956/openreseurope.18327.r36864

**Published:** 2024-02-16

**Authors:** Adnan Asad Karim

**Affiliations:** 1 University of Jaen, Jaen, Spain

The present article has demonstrated an integrated acid leaching-calcination process to valorise post-consumer gypsum from refurbishment and demolition plasterboard waste for producing stuccos. Diluted H _2_SO _4_ acid leaching was used to minimize the water-soluble salt contents and enhance the purity of post-consumer gypsum. Then, the purified post-consumer gypsum was calcined to prepare the stuccos. This work is done by following appropriate methodology, and the results and discussions are described properly with relevant citations. Nevertheless, the authors may choose to include more information in the methodology section about the following issues:

 1) Please justify the reason for selecting a gypsum/solution ratio of 1:3 wt/wt by providing relevant references or preliminary results.

 2) Please briefly elaborate on the method used for semi-quantitative analysis of minerals using XRD peaks.

 The source data underlying the results are provided to ensure full reproducibility. However, I wish to comment here that the authors have given the leaching data and XRD spectra in graphical format only, which is adequate for researchers to understand with the same subject expertise. But to help the other researchers who have no expertise in this subject area, the authors of the present article can also provide raw leaching data and XRD peaks with semi-quantitative analysis in a tabulated form in the Zenodo repository (used by the authors). In addition, the authors should correct typological errors i.e., temperature units in Figure 4, and chemical formulas in Tables 1 and 2.

Is the study design appropriate and does the work have academic merit?

Yes

Is the work clearly and accurately presented and does it cite the current literature?

Yes

If applicable, is the statistical analysis and its interpretation appropriate?

Yes

Are all the source data underlying the results available to ensure full reproducibility?

Yes

Are the conclusions drawn adequately supported by the results?

Yes

Are sufficient details of methods and analysis provided to allow replication by others?

Yes

Reviewer Expertise:

Waste material valorisaton; Biomass; Phosphogypsum; Thermo-chemical processes; Materials characterisation; Bioproducts

I confirm that I have read this submission and believe that I have an appropriate level of expertise to confirm that it is of an acceptable scientific standard.

# Reviewer report: Reviewer response for version 2

**DOI:** 10.21956/openreseurope.18327.r36380

**Published:** 2023-12-15

**Authors:** Said Bouzit

**Affiliations:** 1 Ibn Zohr University of Agadir, Agadir, Morocco

In this work the author presents two types of plaster waste for their recovery. The method and analysis of the results are presented in a clear and structured manner.

 He carried out thermal and structural characterization analyses, but it is necessary to add for information purposes the phase structures of each corresponding change in the peaks!

Is the study design appropriate and does the work have academic merit?

Yes

Is the work clearly and accurately presented and does it cite the current literature?

Yes

If applicable, is the statistical analysis and its interpretation appropriate?

Yes

Are all the source data underlying the results available to ensure full reproducibility?

Yes

Are the conclusions drawn adequately supported by the results?

Yes

Are sufficient details of methods and analysis provided to allow replication by others?

Yes

Reviewer Expertise:

researcher in materials science and engineering

I confirm that I have read this submission and believe that I have an appropriate level of expertise to confirm that it is of an acceptable scientific standard.

# Reviewer report: Reviewer response for version 1

**DOI:** 10.21956/openreseurope.17899.r36002

**Published:** 2023-11-30

**Authors:** Tee How Tan

**Affiliations:** 1 Tunku Abdul Rahman University of Management and Technology,, Kuala Lumpur, Malaysia

The article presents a novel and promising technology for the purification of post-consumer gypsum from plasterboard waste, which could increase its recycling rate and reduce its environmental impact. However, there are some comments that require clarification.

- It would be helpful to provide more information on how to differentiate the RPW and DPW as both of them are collected from recycling centres/sites.
- To enhance readability, it would be helpful to include an appropriate sub-title for each section.
- The article recommends using a 3 wt% H2SO4 solution for 2 hours for industrial-scale leaching purification plant (pg 9). However, the chemical purity produced with this condition did not meet the requirement (Figure 7). Additionally, in conclusion, it mentioned the aim of this work was to develop an acid leaching purification process to achieve consistent chemical purity and CaSO4.2H2O content of >96 wt%.
- The initial setting time for S-GPRW is exceptionally high, 12 mins, compared to others. It would be interesting to know if there are any specific impurities that only exist in GPRW but not GDPW.
- The article lacks in-depth analysis. For instance, it would be helpful to know why water/stucco of S-GPRW and S-GDPW is double than CS and S-MG.

Is the study design appropriate and does the work have academic merit?

Yes

Is the work clearly and accurately presented and does it cite the current literature?

Yes

If applicable, is the statistical analysis and its interpretation appropriate?

Not applicable

Are all the source data underlying the results available to ensure full reproducibility?

Yes

Are the conclusions drawn adequately supported by the results?

Yes

Are sufficient details of methods and analysis provided to allow replication by others?

Yes

Reviewer Expertise:

Sustainable Cement & Concrete Materials, Waste Recycling in Cement & Concrete Materials, Geopolymer

I confirm that I have read this submission and believe that I have an appropriate level of expertise to confirm that it is of an acceptable scientific standard, however I have significant reservations, as outlined above.

# Response

**Published:** 2023-12-06

**Authors:** Miguel Castro-Diaz

**Affiliations:** Loughborough University, Loughborough, England, UK

**1. It would be helpful to provide more information on how to differentiate the RPW and DPW as both of them are collected from recycling centres/sites.** The main difference between these two post-consumer plasterboard wastes is their level of contamination. In page 4 (Materials and methods section), it is indicated that RPW contained 10 wt% of contaminants whilst DPW contained 23 wt% of contaminants. **2. To enhance readability, it would be helpful to include an appropriate sub-title for each section.** The following sub-sections have been included in the following sections of the manuscript: Introduction section: Plasterboard waste generation; Post-consumer gypsum recycling; Aims of this work. Materials and methods section: Plasterboard waste sourcing; Reference materials and chemicals sourcing; Post-consumer gypsum preparation; Laboratory-scale acid leaching post-consumer gypsum purification tests; Experimental techniques. Results and discussion section: Improved mechanical pre-treatment; Acid leaching post-consumer gypsum purification; Properties of stuccos from post-consumer gypsum; Industrial-scale acid leaching post-consumer gypsum purification plant design. **3. The article recommends using a 3 wt% H _2_SO _4_ solution for 2 hours for industrial-scale leaching purification plant (pg 9). However, the chemical purity produced with this condition did not meet the requirement (Figure 7). Additionally, in conclusion, it mentioned the aim of this work was to develop an acid leaching purification process to achieve consistent chemical purity and CaSO4.2H2O content of >96 wt%.** The manuscript recommends the use of a 5 wt% sulfuric acid solution for 1 hour during acid leaching post-consumer gypsum purification, as indicated in the Conclusions section. However, the text may lead to misunderstanding in page 9 and page 10. Accordingly, the following corrections have been made: In page 9, the following sentence has been modified to clarify that the industrial-scale acid leaching purification plant design was based on “the evaluation of” some considerations: “An industrial-scale acid leaching purification plant design for post-consumer gypsum is proposed in this work based on the evaluation of some considerations to reduce economic and environmental impacts.” In page 10, the following sentence has been corrected by removing “for 1 h” since the fourth consideration evaluated acid leaching for 1 hour when using a 5 wt% sulfuric acid solution and for 2 hours when using a 3 wt% sulfuric acid solution: “Batch 2 of GRPW was used to evaluate the fourth consideration using particle sizes < 250 μm at 90 °C for 1 h with stirring at 50 rpm, followed by neutralization.” **4. The initial setting time for S-GPRW is exceptionally high, 12 mins, compared to others. It would be interesting to know if there are any specific impurities that only exist in GPRW but not GDPW.** This following sentence has been included in the manuscript in page 7: “The long setting time of the stucco obtained from the GRPW feedstock (12 minutes) could be due to its low CaSO4.½H2O content (59 wt%) and/or high anhydrous CaSO _4_ content (39.1 wt%) compared to the other stuccos.  However, further research would be required to get a better understanding of this finding, which is outside the scope of this work.  ” **5. The article lacks in-depth analysis. For instance, it would be helpful to know why water/stucco of S-GPRW and S-GDPW is double than CS and S-MG.** The understanding of why the water/stucco ratio of S-GRPW and S-GDPW is double than CS and S-MG is outside the scope of this research. However, some references where similar findings were observed, and potential corrective actions were proposed have been included in the manuscript (Refs. 25-29).
